# Elevated-Temperature Space Charge Characteristics and Trapping Mechanism of Cross-Linked Polyethylene Modified by UV-Initiated Grafting MAH

**DOI:** 10.3390/molecules25173973

**Published:** 2020-08-31

**Authors:** Hong Zhao, Chen Xi, Xin-Dong Zhao, Wei-Feng Sun

**Affiliations:** Key Laboratory of Engineering Dielectrics and Its Application, Ministry of Education, School of Electrical and Electronic Engineering, Harbin University of Science and Technology, Harbin 150080, China; xichen_hrbust@126.com (C.X.); xindong_hrbust@126.com (X.-D.Z.); sunweifeng@hrbust.edu.cn (W.-F.S.)

**Keywords:** cross-linked polyethylene, maleic-anhydride, ultraviolet irradiation, space charge, carrier trap

## Abstract

Space charge characteristics of cross-linked polyethylene (XLPE) at elevated temperatures have been evidently improved by the graft modifications with ultraviolet (UV) initiation technique, which can be efficiently utilized in industrial cable manufactures. Maleic anhydride (MAH) of representative cyclic anhydride has been successfully grafted onto polyethylene molecules through UV irradiation process. Thermal stimulation currents and space charge characteristics at the elevated temperatures are coordinately analyzed to elucidate the trapping behavior of blocking charge injection and impeding carrier transport which is caused by grafting MAH. It is also verified from the first-principles calculations that the bound states as charge carrier traps can be introduced by grafting MAH onto polyethylene molecules. Compared with pure XLPE, the remarkably suppressed space charge accumulations at high temperatures have been achieved in XLPE-g-MAH. The polar groups on the grafted MAH can provide deep traps in XLPE-g-MAH, which will increase charge injection barrier by forming a charged layer of Coulomb-potential screening near electrodes and simultaneously reduce the electrical mobility of charge carriers by trap-carrier scattering, resulting in an appreciable suppression of space charge accumulations inside material. The exact consistence of experimental results with the quantum mechanics calculations demonstrates a promising routine for the modification strategy of grafting polar molecules with UV initiation technique in the development of high-voltage DC cable materials.

## 1. Introduction

With the considerable promotion of developing novel energy in recent years, the clean and renewable electric power generation has become the innovation focus for future power systems [1]. However, it is generally poor in economics and environmental protection that these new decentralized and miniaturized power sources far from the power center, such as solar or wind energy generation, are connected into the power grid by exploiting alternating current (AC) transmission technology. Nowadays, high-voltage direct current (HVDC) transmission technology based on voltage source converter (VSC) has been gradually improved to be capable of fulfilling long distance and large capacity power transmissions [2]. As one of the key equipment to construct DC power grid, cross-linked polyethylene (XLPE) insulated cable is required for higher insulation performances in order to ensure the security and stability of power transmission system [3,4,5]. In spite of high operating temperature, light weight and easy to manufacture, XLPE-insulated HVDC cable is susceptible to the charge injection and space charge accumulation under HVDC electric field, which will lead to electric field distortion, insulation aging, and even to dielectric breakdown. Furthermore, the temperature gradient produced in cable operation will directly cause spatial variation in electrical conductivity of XLPE insulating material, and thus consistently change electric field distribution inside the insulated cable which will in turn influence electrical conductivity distribution by electric heating. Therefore, it is urgent to study the modified polyethylene materials with thermostable dielectric performances.

At present, the modification schemes for XLPE insulation materials are primarily presented by the chemical purity technique, nanocomposite technology, blending and chemical modifications [6]. Especially compared with pure polymer materials, polymer dielectric nanocomposites show various improvements in insulation performances such as the suppressed space charge accumulations, the reduced electrical conductance, and the enhanced breakdown strength, which are attributed to the numerous deep traps introduced by molecular polar groups at nanofiller/matrix interfaces [7,8]. However, this modification mechanism depends greatly on the nanofiller dispersion and the absorbed impurities on nanoparticle surface, which is hardly to be controlled due to the extremely large surface/volume ratio [9,10]. Therefore, it is urgently necessary to develop novel molecular modification strategies to circumvent the inevitable limitation of nanodielectrics. By the first-principles electronic structure calculations, Quirke theoretically studied the energy distributions of the traps deriving from physical and chemical defects, which reasonably suggested that the polar group can render deep traps in polymer materials [11,12]. Zhou reported the excellent electrical properties of chemically modified polypropylene by grafting maleic anhydride and analyzed the underlying mechanisms of space charge suppression, breakdown strength improvement, and conductivity abatement [13]. Zhao developed the chemically modified XLPE by grafting chloroacetic acid allyl ester and correlated space charge accumulation with trapping mechanism to comprehend the achieved insulation performance amelioration [14]. Lee has successfully grafted maleic anhydride (MAH) onto the macromolecular chain of low-density polyethylene by the generally used chemical methods, indicating that the heteropolar space charge accumulations and electrical conductance can be definitely inhibited in LDPE-g-MAH [15]. Although if the chemical grafting and PE cross-linking reactions are expected to occur simultaneously, it is apt to vaporize at high temperature so as to form gas bubbles in equipment pipeline of XLPE cable manufacture due to the low thermogravimetric temperature of MAH, which will intensively deteriorate insulation performances. In an alternative grafting process, which is carried out independently before preparing cross-linkable compounds with the organic-peroxide initiation technique, the grafting and cross-linking reactions cannot be definitely separated when organic-peroxide, MAH, and PE are mixed in the heated parallel twin screws. Even though the reaction rate of grafting MAH onto PE is much higher than that of cross-linking PE molecules at the temperature of DCP initiation, it is difficult to control the actual time of PE molecules staying in mixing chamber. Therefore, gel will be inevitably introduced to form the scorched substance, which is acknowledged as “amber color” of self-generated impurity particles and will severely deteriorate the electrical properties of cable insulation.

In high interest of fulfilling the prospective molecular modification of polymer insulation materials with preferable dielectric properties that can been utilized in industrial HV cable manufactures, it is essentially significant to further develop the molecular-graft modification technology to substitute the traditional chemical schemes. Based on the trap-introducing mechanism of grafting molecules with polar groups, a new tactical scheme of initiating grafting reactions by ultraviolet (UV) irradiation technique is established to eliminate gel generation in practical grafting process, in which the grafting and cross-linking reactions can be definitely demarcated by precisely controlling the power and time of UV incidences. According to the suggested routine, we have prepared cross-linked polyethylene being grafted with MAH (XLPE-g-MAH) utilizing photon-initiator under UV irradiation, and systematically studied the obtained dielectric properties in comparison to the pure XLPE. The fundamental mechanism of space charge suppression is elucidated by analyzing the trap level distributions in XLPE-g-MAH, which accounts for the electric breakdown improvement. Beyond the general experiments of investigating charge injections at a constant ambient temperature, the space charge characterizations at the elevated temperatures increasing to 80 °C have been accomplished, which are essential to correlate molecular properties with space charge formation. Based on density functional theory, the electron bound states intrinsically existing in electronic bandgap of the polyethylene macromolecule being grafted by MAH have been calculated to reveal the physical mechanism of suppressing space accumulations in combination with experimental results.

## 2. Results and Discussion

### 2.1. Molecular Structure Characterization

**Figure 1:**
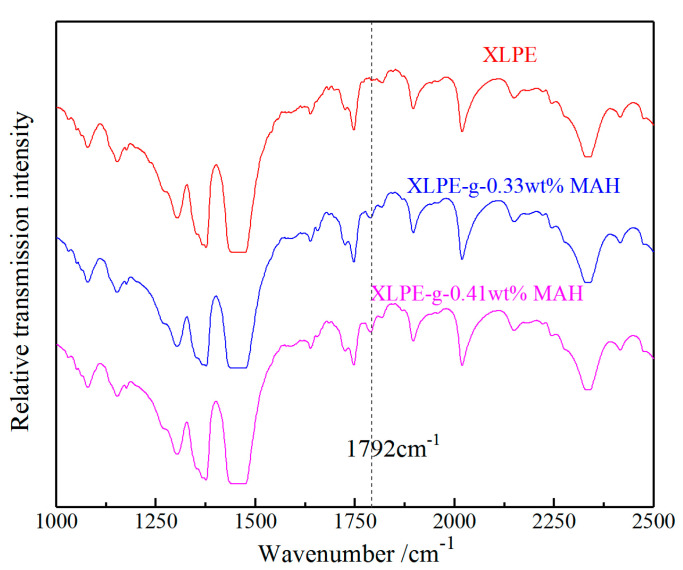
FT-IR transmission spectra of the pure XLPE and the XLPE-g-MAH with 0.33 wt% and 0.41 wt% grafting concentrations.

The grafted MAH on polyethylene molecular chain in XLPE-g-MAH is characterized by FT-IR spectroscopy in comparison to the homologous pure sample (XLPE), which is obtained though the identical preparation process without grafting MAH. The FT-IR spectroscopy results shown in Figure 1 verify the changing molecular structures caused by UV-initiated grafting reactions. The carbonyl (C=O) of MAH molecules grafted onto polyethylene molecular chains can be identified by the characteristic stretching vibration peak at 1792 cm^−1^ in IR transmission spectra of the XLPE-g-MAH samples, which have been hot-degassed to eliminate ungrafted MAH and cross-linking by-products. Especially, the intensity of the newly arising C=O peak for XLPE-g-MAH increases with the MAH grafting concentration, to the highest value for XLPE-g-0.41 wt%MAH. In contrast, the IR absorption peak at 1792 cm^−1^ deriving from C=O of MAH molecule cannot be presented by pure XLPE without MAH-grafting reaction, confirming that the MAH groups bonding to polyethylene molecular chains have been acquired in XLPE-g-MAH samples. The FT-IR spectroscopy results explicitly indicate that the MAH molecules have been successfully grafted onto XLPE molecules for each grafting concentration.

### 2.2. Space Charge Characteristics

**Figure 2:**
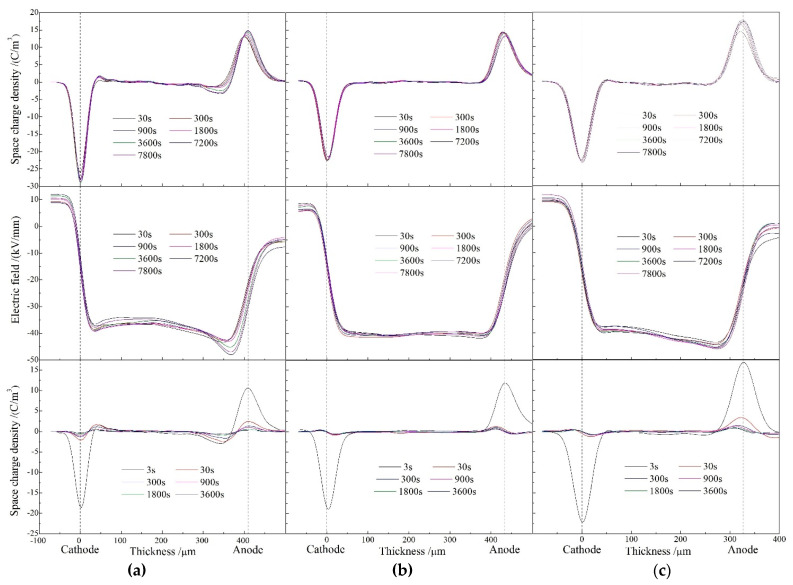
Space charge distributions at 25 °C in (**a**) XLPE, (**b**) XLPE-g-0.33wt%MAH, and (**c**) XLPE-g-0.41wt%MAH under applied DC electric field 40 kV/mm (upper panels) and in short-circuit (bottom panels). Electric field distributions across samples in polarization process are illustrated in middle panels.

Space charge distributions of XLPE and XLPE-g-MAH at various temperatures from 25 to 80 °C are tested with pulsed electroacoustic (PEA) method to comprehensively reveal the underlying physics of charge injections. Figure 2 illustrates the space charge accumulation and dissipation under the applied DC electric field of 40 kV/mm for 130 min and then in short-circuit at the temperature of 25 °C. It is indicated from Figure 2a that heterocharges have been evidently accumulated near both cathode and anode due to impurity ionization in pure XLPE, with the space charge density increasing with polarization time to the highest peak value of 3 C/m^3^. The substantial heterocharges injected in pure XLPE strengthen the electric field near electrodes and thus exacerbate electric field distortion, as the electric field distribution across sample shown in the middle panel of Figure 2a, implying that the peak electric field at the positive electrode approaches to 47 kV/mm. In contrast, no obvious charge injection has been found at both electrodes in XLPE-g-MAH samples even after polarizing for 130 min as shown in upper panels of Figure 2b,c, without charge accumulation being observed in short-circuit as shown in the bottom panels. The internal electric field distortions of XLPE-g-MAH materials are alleviated with the peak electric fields being restricted below 41 and 44 kV/mm, respectively.

**Figure 3:**
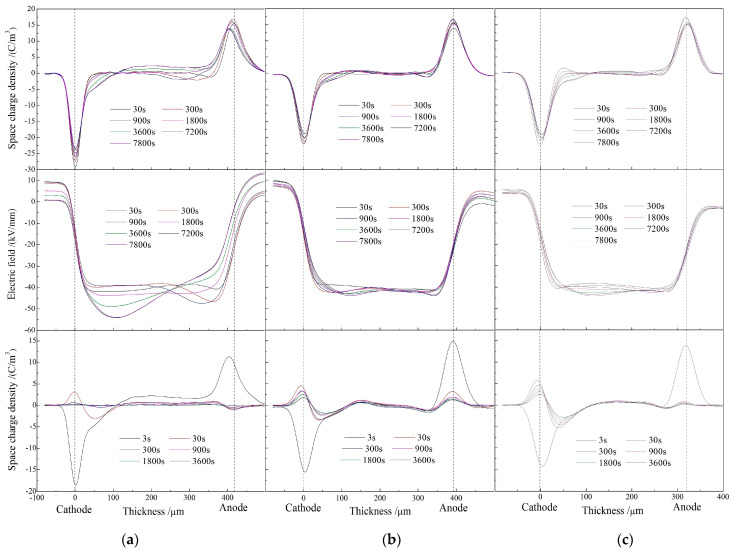
Space charge distributions at 60 °C in (**a**) XLPE, (**b**) XLPE-g-0.33wt%MAH, and (**c**) XLPE-g-0.41wt%MAH under applied DC electric field 40 kV/mm (upper panels) and in short-circuit (bottom panels). Electric field distributions across samples in polarization process are illustrated in middle panels.

When the test temperature is raised to 60 °C, the heterocharges arise near the anode and a large number of negative homocharges also accumulate near cathode in XLPE after being polarized for 130 min, as shown in left panel of Figure 3a. With the increase of polarization time under DC electric field, the heterocharge injection gradually transforms to the negative homocharge injection, indicating that the positive homocharges injected from anode are neutralized by the increasing negative heterocharges when the injected charges penetrate into internal region to form the positive space charge accumulations. After being short-circuit for 60 min, the homocharges are found near cathode with the peak charge density approaching to 3.14 C/m^3^, while only a small amount of positive homocharges are detected near anode. The peak electric field at the negative electrode reaches to 55 kV/mm in XLPE after applying the electric field for 130 min, leading to the electric field distortion of 37.5% as shown in middle panels of Figure 3a. Although the total charge quantity and charge density peak of the homocharges near the negative electrode in XLPE-g-0.33wt%MAH are similar to those of XLPE, the short injection distance and the minimal positive heterocharges near anode even after being polarized for 130 min verify the evident improvement in space charge characteristics of XLPE-g-MAH. Further, only the homocharges are injected near cathode for XLPE-g-0.33wt%MAH with a short injection distance of 70 μm, resulting in slightly distorted electric field of 43 kV/mm. For XLPE-g-0.41wt%MAH, a few positive heterocharges appear near cathode at the initial stage of electric polarization and then, vanish with the increasing polarization time, in contrast to the appreciable increment of homocharge injections. It is thus reasonably conjectured that positive heterocharges injected at the initial stage of applying electric field can induce negative homocharges near the cathode, leading to further increased electric field of 44 kV/mm by 20% distortion near the cathode. Complying to the Schottky injection mechanism, the homocharge injection is intensified and eventually accumulate near cathode after completely neutralizing positive charges.

**Figure 4:**
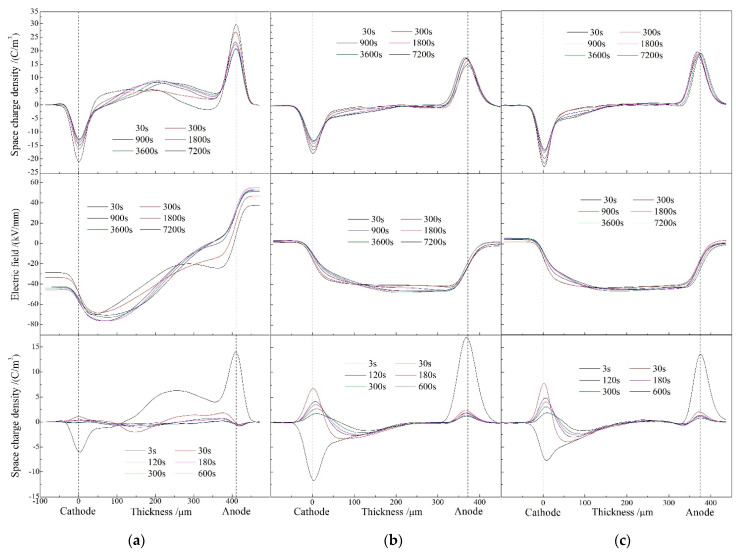
Space charge distributions at 80 °C in (**a**) XLPE, (**b**) XLPE-g-0.33wt%MAH, and (**c**) XLPE-g-0.41wt%MAH under applied DC electric field 40 kV/mm (upper panels) and in short-circuit (bottom panels). Electric field distributions across samples in polarization process are illustrated in middle panels.

The space charge distributions at the temperature of 80 °C under the applied electric field of 40 kV/mm for representative polarization time of 120 min and short-circuit time 10 min are presented in Figure 4. During the polarization process of XLPE, a considerable amount of homocharges are accumulated near both cathode and anode with the apparently high density of space charge approaching to the peak value of 10 C/m^3^ inside material. The charge injection at cathode and anode permeates to 100 and 200 μm distances inside XLPE, respectively. The electric field in XLPE has been 95% distorted to the peak value of 78 kV/mm under the electric field being only applied for 120 min. In comparison, the homocharges injected in XLPE-g-0.33wt%MAH are distributed across a smaller area of 0–150 μm near cathode with a smaller peak of 4.8 C/m^3^, while no conspicuous charge accumulation occurs in vicinity of anode. It is confirmed from short-circuit charge distributions of Figure 4c that the space charge accumulations of XLPE-g-0.41wt%MAH distribute in the smallest region of 0–100 μm away from both electrodes with a slightly lower charge density peak of 4.7 C/m^3^. Accordingly compared to XLPE, the electric field distortions of XLPE-g-0.33wt%MAH and XLPE-g-0.41wt%MAH recede by ~40% with the peak amplitudes being limited to 47 and 46 kV/mm, respectively.

In summary for this section, we can draw the conclusion that the space charge characteristics have been appreciably improved so as to alleviate the corresponding electric field distortions near electrodes in XLPE-g-MAH materials compared with pure XLPE material in the whole temperature range of cable operation. The space charges in higher density deriving from the charge-captures by grafting-introduced deep traps only accumulate in a very thin layer region near electrodes. It will not produce large electric field distortion in modified materials even at 80 °C, due to the electrostatic shielding layers near electrodes, which originate from the fixed and densely distributed charges that have been captured into the grafting-introduced deep traps [14]. It is also proved that the increment of grafting concentration can promote the inhibition of space charge such that the charge injection density and electric field distortion of XLPE-g-0.41wt%MAH are lower than those of XLPE-g-0.33wt%MAH.

### 2.3. Electric Charge Traps

The space charge accumulation and charge transport can be essentially correlated to the charge carrier trapping behavior [16]. In order to comprehend the space charge suppression in XLPE-g-MAH, the energy distributions of charge traps have been precisely evaluated from TSC measurements according to the modified TSC theory [17]. The TSC testing temperatures from −30 to 170 °C cover the thermal excitation energies of the charges captured by both intrinsic traps of XLPE and grafting-introduced deep traps, in which the practical operating temperatures of HVDC cable are completely encompassed. TSC test characterizes the current produced by the thermally excited charges, which have been trapped by bound states of electron or holes, in which the intensity and position of current peak signify the quantity of the trapped charges with a specific thermal excitation energy (the trapping level depth) [17]. Accordingly, the new peak in TSC spectra of XLPE-g-MAH materials arising at a higher temperature identifies a deeper tapping level, while the lower temperature peak corresponds to the intrinsic traps from structural defects of XLPE matrix.

**Figure 5:**
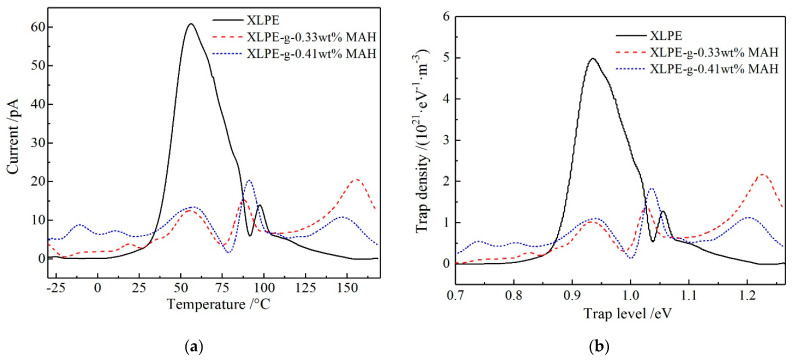
(**a**) TSC temperature spectra and (**b**) trap level distributions of XLPE and XLPE-g-MAH.

The general peaks arising in the temperature range of 30–90 °C as shown in Figure 5a originate from the detrapping of the captured charges in the intrinsic shallow traps of structural defects between lamellae in XLPE matrix, which is caused by the thermal relaxations of XLPE molecules and dominates the quantity of space charges (integral of peak curve). At the temperature of about 100 °C, both XLPE and XLPE-g-MAH represent a narrow low-amplitude peak of pyroelectric current, which derives from the annihilating structural defects when XLPE crystalline phase is completely melted. In particular, at the temperature of 150 °C, a characteristic peak with a low-amplitude and a small integral area appears in XLPE-g-MAH, which is caused by charge detrapping from the deep traps introduced by grafting MAH onto XLPE molecular chain. Employing the method presented by the reference [18], the trap level depths versus temperature are calculated from the TSC results of Figure 5a, with the results being shown in Figure 5b. The energy level depth of the deep traps introduced by grafting MAH approaches to 1.2 eV, which agrees well with the theoretical results of the first-principles calculations. Since carriers are more likely to be captured into deep traps than shallow traps, there are not enough carriers being trapped into the intrinsic shallow traps of the structural defects in XLPE matrix when applying a high-voltage electric field to the modified materials of XLPE-g-MAH. Therefore, the amplitude and integral area of the pyroelectric peak at 30–90 °C are obviously smaller than that of pure XLPE, accounting for the greatly reduced space charge accumulation in XLPE-g-MAH samples.

**Figure 6:**
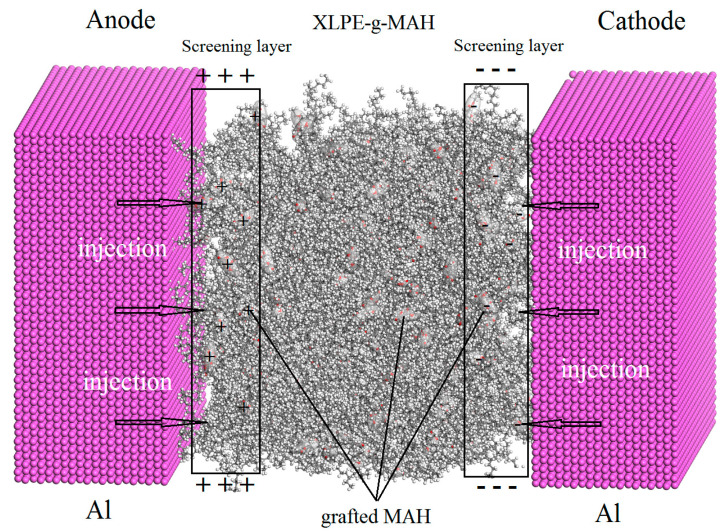
Schematic Coulomb-potential screening mechanism of suppressing space charge accumulation inside XLPE-g-MAH under DC electric field.

It is illustrated from the measured TSC spectra and the derived trap level distributions of XLPE and XLPE-g-MAH as shown in Figure 5, the magnificent deeper traps have been introduced by grafting MAH, the current and density peaks of which are apparently higher for the larger grafting content. It also noted that the grafting-introduced traps are slightly deeper for the higher grafting concentration. The deepest trap level peaks of XLPE, 0.33 wt%, and 0.41 wt% XLPE-g-MAH are 0.95, 1.21, and 1.25 eV with trap level densities of 4.97, 2.17, and 1.13 (10^21^·eV^−1^·m^−3^), respectively. The homogeneously distributed deep traps in high density being fixed on the grafted MAH will effectively capture the injected heterocharges and thus forms a charged Coulomb-potential screening layer near electrodes under DC electric field, which can block further charge injections and impede carrier transports. Hence, the space charge accumulations in XLPE-g-MAH is remarkably suppressed due to the deep traps introduced by grafting MAH onto polyethylene molecules, as schematically shown in Figure 6. Furthermore, the graft-introduced traps being 0.3 eV deeper than intrinsic traps can retain highly efficient suppression mechanism of space charge accumulation at the elevated temperatures approaching to 150 °C, which is significantly higher than 55 °C for pure XLPE. These results suggest that XLPE-g-MAH can persist in high dielectric performances through the charge trapping mechanism at high temperatures rising to 150 °C.

**Figure 7:**
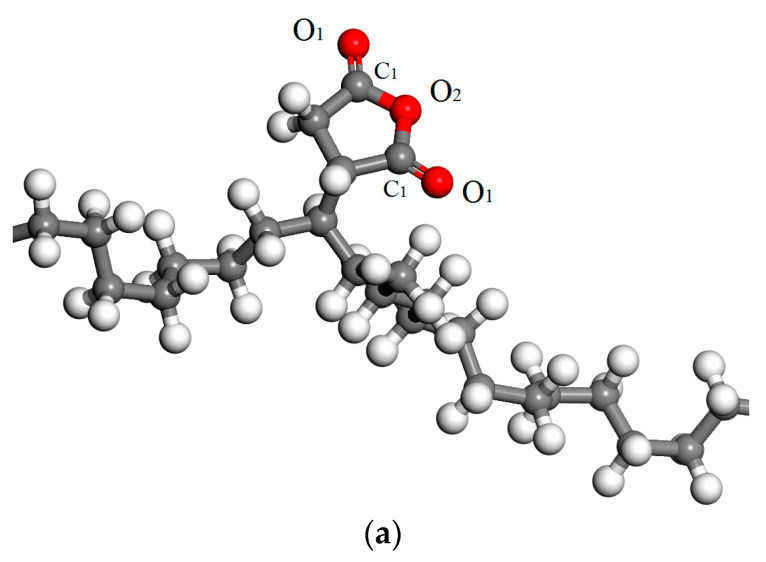

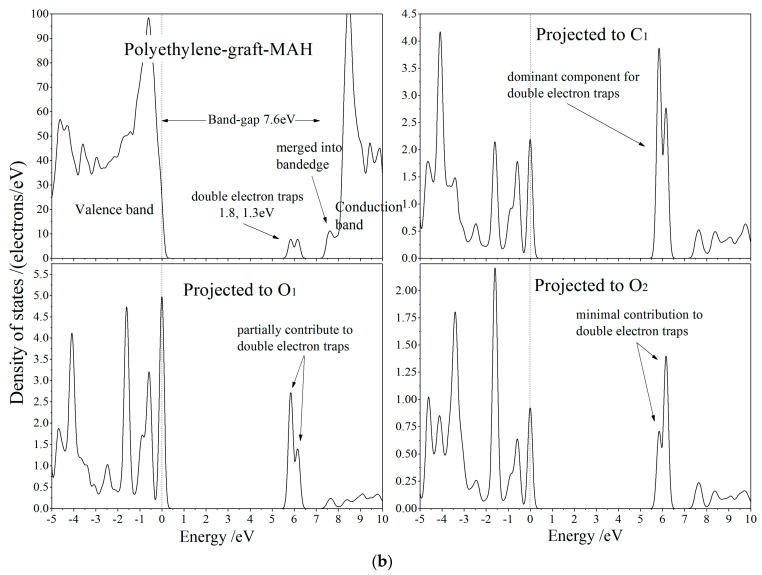
(**a**) Schematics of grafting MAH to polyethylene molecule with the carbon and oxygen atoms of carbonyl and the ester oxygen atom being identified by C_1_, O_1_, and O_2_, respectively and (**b**) the total and atomic-projected density of states for PE-g-MAH from first-principles calculations. The highest occupied molecular orbital (HOMO) level is set to be the energetic zero, as indicated by the vertical dashed line.

Energy levels of deep traps in XLPE-g-MAH materials have been derived from the TSC spectra to be about 1.25 eV. This result is consistent with the density of states (DOS) from the first-principles electronic structure calculations, as shown in Figure 7 for the relaxed (geometrically optimized) model and the total/projected DOS of the polyethylene molecule grafted by MAH (PE-g-MAH). It could be verified by the DOS results of Figure 7b that the background electronic states presented by polyethylene molecule in 30 polymeric degree form electronic bands in comparison to the local states confined in a few specific energy levels with a relatively minimal DOS values. The grafted MAH can introduce two unoccupied electronic bound states in the bandgap of polyethylene molecule, which will serve as the deep traps of free electrons in conduction band with the energy level of 1.3 and 1.8 eV reference to conduction band minimum (CBM). Very close to CBM, an unoccupied bound state of a little lower energy level is also introduced by the grafted MAH, which has merged into conduction band to become a new CBM, resulting in 0.4 eV reduction of bandgap.

The projected density of states (Figure 7b) shows that the two trap states are mainly derived from the carbonyl-carbon atom (C_1_) and a small fraction from the carbonyl-oxygen atom (O_1_) of the grafted MAH, where the electron wave functions are distributed in local space around the carbonyl-bond (C=O). Two adjacent C=O bonds in the grafted MAH overlap and result in two unoccupied splitting molecular orbitals as the bound states, which can capture electrons in conduction band. The trap states of 1.8 eV are composed of 60% C_1_ and 39% O_1_ atomic orbitals, while the traps of 1.3 eV mainly come from C_1_ atomic orbitals (>70%) with a minor part (<30%) from atomic orbitals of O_1_ and O_2_ (ester oxygen). The electronic structure results of theoretical calculations being consistent with experimental results of TSC further prove that the anhydride group of the grafted MAH can introduce electronic states of deep traps with energy levels among bandgap of polyethylene, which will efficiently scatter and capture electrons in conduction band. In supplement, the coupling of the anhydride groups on the grafted MAH splits the degenerate electronic states into a couple of electron deep traps, resulting in a deeper trap of 1.8 eV that cannot be verified by TSC experiments unless the testing temperature is raised to higher than ~300 °C which goes far beyond the operation temperature and exceeds the melting point of XLPE.

## 3. Materials and Methods 

### 3.1. Material Preparation

In order to promote the preparation efficiency of grafted materials, the master-batches with high grafting content are first prepared by grafting MAH to low-density polyethylene (LDPE). The basic raw materials employed in master-batch preparations are presented as follows: LDPE (LD200GH, Sinopec Co. Ltd., Beijing, China) as basis material, benzophenone (BP, Jinleiyuan Chemical Co. Ltd., Lianyungang, China) for initiating grafting reaction under photon irradiation, and maleic anhydride (MAH, Ruierfeng Chemical Co. Ltd., Guangzhou, China) as the grafting reactant. In the melting blend process of preparing initial mixing materials, the pristine LDPE materials are melted at 120 °C temperature with a rotating speed of 60 rpm for 1 min in a torque rheometer (RM200C, Hapro Co. Ltd., Harbin, China), and then 3 wt% MAH and 0.2 wt% BP are added to be blended for 4 min and cooled down to room temperature to obtain the uniformly mixing compounds. For photon-initiated grafting reactions, the prepared blends are first treated in a flat vulcanizer at 120 °C with the pressure being raised by 5 MPa per 5 min from 0 to 15 MPa to make the material melt. Especially, the melt materials are uniformly poured into the plate mold on irradiation platform, which was transferred at a constant speed though a light source array of UV LED units (NVSU233A-U365, Riya Electronics Chemistry Co. Ltd., Shanghai, China) for 10 s at normal pressure and room temperature in air atmosphere. Finally, the MAH-grafted master-batches (LDPE-g-MAH) are prepared after being degassed at 80 °C for 48 h in a vacuum oven to remove residual BP and ungrafted MAH molecules. By means of gel extraction experiments, the gel contents of LDPE-g-MAH master-batches prepared by UV grafting technique are tested to be <0.01%, which are considerably lower than those polyethylene materials modified by the chemically grafting methods. Because 0.01% is the accuracy limitation of gel extraction experiments, the gel content could be regarded as zero. The concentrations of the grafted MAH in LDPE-g-MAH master-batches are determined by chemical titration to be 1.64%.

In the second step, the LDPE-g-MAH master-batches need to be mixed with LDPE in an appropriate proportion so that the MAH content was diluted to a reasonable level, which could effectively inhibit space charge accumulations. Accordingly, the necessary processes of cross-linking polyethylene molecules are carried out by melt mixing pure LDPE and LDPE-g-MAH master-batches (as the blending components listed in Table 1) with 2 wt% dicumyl peroxide (DCP, Nobel Co. Ltd., Aksu, China) as the cross-linking agent and 0.3 wt% pentaerythritol ester antioxidant (Irganox1010, BASF SE., Ludwigshafen, Germany) at 120 °C for 5 min in the torque rheometer with a rotating speed of 60 rpm. In order to fully accomplish cross-linking reactions, the obtained compounds are further processed under 15 MPa at 175 °C for 1 h in the flat vulcanizer and then cooled down to ambient temperature at a rate of 25 °C/min under 15 MPa in a cooler. The electrically modified XLPE-g-MAH materials are eventually achieved after being hot-degassed for 48 h at 80 °C in a vacuum oven to eliminate internal stresses and remove reaction by-products.

### 3.2. Characterization and Testing Methods

Infrared absorption spectra of characterizing the grafted MAH in modified materials are tested for a film sample in 0.3 mm thickness by Fourier-transform infrared (FT-IR) spectrometer (FT/IR-6100, Jiasco Trading Co. Ltd., Shenyang, China) with a wave-number range of 500–4000 cm^−1^ being scanned in the resolution of 2 cm^−1^. Employing pulsed electroacoustic (HY-PEA-DPT01, HeYi Electric Co. Ltd., Shanghai, China) system, the space charge characteristics are measured at various temperatures of 25–80 °C under electric field of 40 kV/mm in polarization for 130 min and short-circuit for 60 min, in which the tested materials are required to be fabricated into 50 × 50 × 0.3 mm^3^ square film samples with both sides being vacuum evaporated by aluminum film electrodes in 25 mm diameter. Thermal stimulation currents (TSC, Harbin University of Science and Technology, Harbin, China) are tested to analyze the energy level distribution of charge traps in materials. The samples in 100 µm thickness are first applied with the electric field of 40 kV/mm at 60 °C for 30 min, through which carriers have been injected from electrodes and captured by charge traps to form space charge accumulations in the insulating material. Then, the sample-electrode testing system is rapidly cooled by liquid nitrogen down to −30 °C being stabilized for 10 min, in which the samples were in short-circuit with the applied voltage being removed. The pyroelectric discharge currents of the short-circuiting samples are continuously measured by a microcurrent meter when temperature is increased from −30 to 170 °C with a heating rate of 3 °C/min.

### 3.3. Molecular Model and Calculation Methodology

Molecular model for the symptomatic polyethylene molecule grafted by a MAH (PE-g-MAH) molecule is initially constructed with random distributed torsion according to the equilibrium C-C and C-H bond lengths of 1.50 and 1.10 Å respectively, in which the PE molecule of 30 polymerization degree is grafted by MAH molecule near the middle position of PE backbone chain based on rotational isomeric state (RIS) model. Polyethylene molecule in 30 polymeric degree could acquire enough long molecular chains to present a background electronic structure of polyethylene that the electronic waves extending throughout the whole polyethylene molecule can form a band by a huge number of such electrons. Accordingly, the electronic states introduced by grafting MAH could be regard as additional localized electronic components which will not return to effect the electronic states of polyethylene. The constructed initial polymer configurations are geometrically optimized to a relaxed structure by total energy functional minimization with conjugated gradient algorithm in first-principles calculations [19], by which the energy change, atomic force, and displacement are theoretically evaluated to be lower than 1.0 × 10^−5^ eV/atom, 0.03 eV/Å, and 0.001 Å respectively. The electronic structures are calculated for molecular orbitals and electronic density of states to investigate band-edge features and grafting-introduced local states (carrier traps). The first-principles calculations are performed employing the scheme of all-electron and numerical atom orbitals [20,21], as implemented in DMol3 program of Materials Studio 8.0 software package (Accelrys Inc., Materials Stutio v8.0.0.843, San Diego, CA, USA). The detailed methodology adopted in calculations is listed in Table 2.

## 4. Conclusions

Space charge characteristics of XLPE dielectrics at elevated temperatures have been significantly improved by utilizing UV initiation technique in MAH grafting process, the underlying modification mechanisms of which are systemically investigated by TSC analyses in combination with quantum mechanics calculations. XLPE-g-MAH materials with almost zero gel content have been synthesized through the successful UV initiated reactions of grafting MAH onto polyethylene molecules, which has been verified by the gel extraction experiments and IR spectroscopic characterizations. The XLPE-g-MAH materials acquire excellent DC dielectric properties, which have been essentially elucidated by analyzing space charge distributions at various temperatures for cable operation. The modified dielectric properties of XLPE-g-MAH are demonstrated to be attributed to the double deep traps deriving from the coupling anhydride group of the grafted MAH. Space charge injection can be remarkably impeded by Coulomb-potential screening from the charges captured by deep traps near electrodes. This study suggests a strategic scheme of graft modification for realizing high performance insulating materials, which is prospective to be feasibly applied in HVDC cable manufactures.

## Figures and Tables

**Table 1:** Blending components (wt%) for preparing XLPE and XLPE-g-MAH samples.

Samples	LDPE	LDPE-g-MAH Master-Batches	Grafting Content of MAH
XLPE	100	0	0
XLPE-g-0.41wt%MAH	75	25	0.41
XLPE-g-0.33wt%MAH	80	20	0.33

**Table 2:** Schemes and parameters adopted in the first-principles calculations by DMol3.

Electronic Hamiltonian	Scheme	Condition and Parameter
Exchange-correlation	Meta-GGA	M11-L [19]
Integration accuracy	Grid	2000 points /atom
SCF	Tolerance	1 × 10^−6^ eV /atom
	Multipolar expansion	Octupole
	Charge density mixing	Charge = 0.3, DIIS = 5
Core treatment	All electron	
Numerical basis set	DNP	Basis file 4.4
Orbital cutoff	Global	5.0 Å
